# Application of Phosphoproteomics to Find Targets of Casein Kinase 1 in the Flagellum of *Chlamydomonas*

**DOI:** 10.1155/2012/581460

**Published:** 2012-12-18

**Authors:** Jens Boesger, Volker Wagner, Wolfram Weisheit, Maria Mittag

**Affiliations:** Institute of General Botany and Plant Physiology, Friedrich Schiller University Jena, Am Planetarium 1, 07743 Jena, Germany

## Abstract

The green biflagellate alga *Chlamydomonas reinhardtii* serves as model for studying structural and functional features of flagella. The axoneme of *C. reinhardtii* anchors a network of kinases and phosphatases that control motility. One of them, Casein Kinase 1 (CK1), is known to phosphorylate the Inner Dynein Arm I1 Intermediate Chain 138 (IC138), thereby regulating motility. CK1 is also involved in regulating the circadian rhythm of phototaxis and is relevant for the formation of flagella. By a comparative phosphoproteome approach, we determined phosphoproteins in the flagellum that are targets of CK1. Thereby, we applied the specific CK1 inhibitor CKI-7 that causes significant changes in the flagellum phosphoproteome and reduces the swimming velocity of the cells. In the CKI-7-treated cells, 14 phosphoproteins were missing compared to the phosphoproteome of untreated cells, including IC138, and four additional phosphoproteins had a reduced number of phosphorylation sites. Notably, inhibition of CK1 causes also novel phosphorylation events, indicating that it is part of a kinase network. Among them, Glycogen Synthase Kinase 3 is of special interest, because it is involved in the phosphorylation of key clock components in flies and mammals and in parallel plays an important role in the regulation of assembly in the flagellum.

## 1. Introduction

Eukaryotic cilia or flagella are microtubule-based organelles that are highly conserved in protein composition and structural organization from protozoa to mammals. They are structurally characterized by nine microtubular doublets surrounding two central microtubular singlets [1]. Substructures like dynein arms and radial spokes are associated with the axoneme and important for motility in the flagellum. Matrix proteins that are not tightly associated with the flagellar membrane or the axoneme serve diverse functions in the flagellum and can be involved in intraflagellar transport [2].

Since many years, the green biflagellate alga *Chlamydomonas reinhardtii*, whose genome has been sequenced, is used as a model to study flagella structure, assembly, formation, and motility [3]. *C. reinhardtii* uses flagella for motility in aqueous environments, for attaching to surfaces and for cell-cell recognition during mating. A proteomic analysis of *Chlamydomonas* flagella revealed more than 600 proteins [4] that include, for example, motor and signal transduction components as well as proteins with homologues associated with human diseases (e.g., polycystic kidney disease, retinal degeneration, hydrocephalus, or changes in the left-right symmetry of organs) collectively known as ciliopathies [5]. But in many cases, Flagellar Associated Proteins (FAPs) still have unknown function. 

Among the proteins in the flagellum, 21 protein kinases and 11 protein phosphatases were found pointing to regulation by reversible protein phosphorylation in this organelle. Phosphorylation events on specific amino acids residues can affect protein function, its intracellular localization, its activity, and its affinity to interaction partners (for review see [6]). But the identification of substrates for kinases in the phosphoregulatory pathway is still a challenge. In *C. reinhardtii*, several proteomes and phosphoproteomes of subcellular compartments (reviewed in [7, 8]) were analyzed including environmentally modulated photosynthetic membranes [9], the eyespot [10], and the flagellum [11]. The flagellum phosphoproteome was first studied under physiological conditions without postincubation of isolated flagellar proteins with ATP to increase the phosphorylation status. 126 *in vivo* phosphorylation sites were found belonging to 32 different structural and motor proteins, several kinases, and proteins with protein interaction domains [11]. Furthermore, a dynamic phosphorylation pattern and clustering of phosphorylation sites were found in some cases, indicating the specific control of proteins by reversible phosphorylation in the flagellum. In another study, flagellum phosphoproteins were examined during flagella shortening. In this case, postincubation with ATP was undertaken. Thereby, half of the identified phosphoproteins were only detected in shortening flagella [12]. 

The axoneme of *Chlamydomonas* flagella anchors multiple inner arm dyneins and a network of kinases and phosphatases that control motility by reversible protein phosphorylation [13]. One of the involved flagellum kinases is Casein Kinase 1 (CK1) [14–16]. In pharmacological experiments using a specific CK1 inhibitor (CKI-7), it was shown that CK1 regulates dynein activity and flagellum motility by phosphorylation of the Inner Dynein Arm I1 Intermediate Chain 138 (IC138) [14, 15]. Moreover, silencing of CK1 results in alterations of circadian phototaxis (shortening of the period), defects in flagella formation, and in hatching of the daughter cells [17]. Interestingly, alterations in the expression of several other key players of the clock machinery of *C. reinhardtii* named Rhythm of Chloroplast (ROC) and a homologue of Constans (CrCO) have in parallel severe effects on hatching, flagella formation, and/or movement, underlining that these processes are interconnected in *C. reinhardtii *[17–19]. 

Regarding the multiple functions of CK1 in flagella formation and motility along with its regulatory role in the circadian system in *C. reinhardtii*, we were interested in the identification of CK1 targets in flagella beside IC138. In a comparative phosphoproteomic approach using wild-type cells with and without CKI-7 treatment, we determined the targets of CK1 in the flagellum. In the CKI-7-treated cells, several phosphoproteins were missing or were identified with a reduced number of phosphorylation sites, compared to untreated wild-type cells. Also novel phosphopeptides or additional phosphorylation sites of known phosphopeptides were identified in the CKI-7-treated cells, suggesting that CK1 is part of a signaling network in the flagellum.

## 2. Materials and Methods

Standard molecular biology methods were done according to [20].

### 2.1. Cell Culture

*C. reinhardtii *strain 137c (*nit1 nit2*) was used with whom the flagellar proteome and phosphoproteome were analyzed [4, 11]. Cells were grown in TAP medium [21] under a 12 h light-12 h dark cycle (LD 12 : 12) with a light intensity of 71 *μ*E m^−2^ sec^−1^ (1 E = 1 mol of photons) at 24°C. The beginning of the light period is defined as time zero (LD0) and the beginning of the dark period is LD12. In some cases, cells were released after growth in LD into constant conditions (LL) of dim light (15 *μ*E m^−2^ sec^−1^). 

### 2.2. Crude Extract Preparation and Immunodetection

Protein extracts were prepared as described previously [11]. The concentration of proteins was measured according to [22]. Immunoblots were done with antibodies against phosphoSer (Qiagen) and phosphoThr (Cell Signaling Technology) according to the manufacturer's instructions. Polyclonal antibodies against the C-terminal part of CK1 (amino acids 131–333 out of 333; ID JGI Vs3: 137286) were also used [23]. For this, the C-terminal part of CK1 was expressed and purified from *E. coli* according to the Qiagen protocol. Antibodies were raised by the “Pineda-Antikörper-Service,” Berlin, Germany. Immunoblots were done as described [11] using the polyclonal anti-CK1 antibody in a dilution of 1 : 5,000. 

### 2.3. Densitometry Analysis

Quantifications were done with the Image Master 2D Elite (version 4.01) software from GE Healthcare (formerly Amersham Pharmacia Biotech). 

### 2.4. Measurement of Swimming Velocity of *C. reinhardtii* Cells

Measurement of swimming velocity was done by using a hemocytometer and a differential interference contrast microscope with a total magnification of 400 including a personal computer with a digital video recording system to measure displacement versus time. The swimming velocity was determined manually by measuring the linear displacement of cells on the scale of the micrometer. 10 samples were measured to obtain the average velocity of a given sample.

### 2.5. Cell Growth, CKI-7 Treatment, Isolation of Flagella, Protein Digestion, and Enrichment of Phosphopeptides by Immobilized Metal Affinity Chromatography (IMAC)

Cells were grown in a LD cycle and harvested at the end of the night (LD24) at a cell density of 2-3 × 10^6^ cells mL^−1^ by centrifugation (700 ×g, 5 min, 4°C).Cells were resuspended in one-half volume of minimal medium [21] and then the culture was kept under dim light conditions for 29 h representing subjective day (LL29), before cells were harvested (700 ×g, 15 min, 4°C). In some cases, the CK1 inhibitor, CKI-7, (N-(2-Aminoethyl)-5-chloroisoquinoline-8-sulfonamide; Toronto Research Chemicals Inc.) [24], was added to the culture to a final concentration of 50 *μ*M following the shift to LL conditions. Isolation of the matrix membrane axoneme fraction (MMA) of flagella, tryptic digestion of MMA proteins, and enrichment of flagellum phosphopeptides by IMAC were done as previously described [11]. 

### 2.6. Peptide Identification by Nano-Liquid Chromatography-Electrospray Ionization-Mass Spectrometry (nLC-ESI-MS) and Data Analysis

nLC-ESI-MS and data analysis were carried out as described before [11]. Briefly, phosphopeptides were subjected to nLC-ESI-MS using an UltiMate 3000 nano-HPLC (Dionex Corporation) with a flow rate of 300 nL min^−1^ coupled online with a linear ion trap ESI-MS (Finnigian LTQ, Thermo Electron Corp.). The instrument was run by the data-dependent neutral loss method, cycling between one full MS and MS/MS scans of the four most abundant ions. After each cycle, these peptide masses were excluded from the analysis for 10 sec. The detection of a neutral loss fragment (98, 49, or 32.66 Da) in the MS^2^ scans triggered an MS^3^ scan of the neutral loss ion representing the dephosphorylated peptide. 

Data analysis was done using the Proteome Discoverer software (Version 1.0) from Thermo Electron Corp. including the SEQUEST algorithm [25]. The software parameters were set to detect a modification of 79.96 Da in Ser, Thr, or Tyr in MS^2^ and MS^3^ spectra. For the database searches with MS^3^ data, modifications of −18.00 Da on Ser and Thr residues representing the neutral loss were additionally used. Further, detection of a modification of 16 Da on Met representing its oxidized form was enabled and carboxyamidomethylation of Cys residues was enabled as a static modification. Peptide mass tolerance was set to 1.5 Da in MS mode. In MS^2^ and MS^3^ modes, fragment ion tolerance was set up to 1 Da. The parameters for all database searches were set to achieve a false discovery rate (FDR) of not more than 1% for each individual analysis. Data were searched against the flagellar proteome database [4] (http://labs.umassmed.edu/chlamyfp/index.php). Additionally, NCBI and the Joint Genome Institute *C. reinhardtii* databases (Version 2 and Version 3) were used for data evaluation.

## 3. Results 

### 3.1. The Effects of the CK1 Inhibitor CKI-7 on the Phosphorylation Pattern of Flagellum Proteins and the Swimming Velocity of *C. reinhardtii*

**Figure 1:**
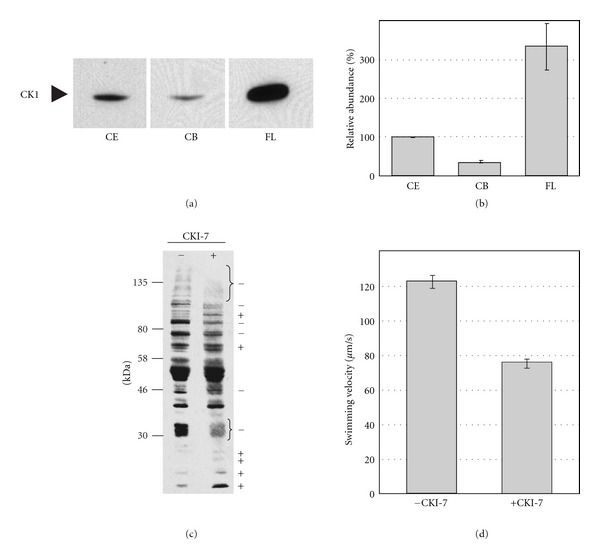
Enrichment of CK1 in flagella and the influence of CK1 inhibition on the phosphorylation status of flagellum proteins and swimming velocity of *C. reinhardtii* cells. (a) Cells were grown in TAP in a 12 h light-12 h dark cycle and then released to dim light (LL) according to Section 2. Cells were harvested at LL29 and flagella were isolated and a whole cell crude extract (CE), a flagellar extract (FL), and an extract from cell bodies lacking flagella (CB) were prepared. 25 *μ*g proteins per fraction were separated by SDS-PAGE and analyzed by immunoblotting with anti-CK1 antibodies according to Section 2. (b) For quantifications, the amount of CK1 detected in the whole cell crude extract was set to 100%. Quantifications were done with three biological replicates using the ImageMaster 2D Elite Vs.4.01 software (GE Healthcare). (c) Changes in the phosphorylation pattern of flagellum proteins in cells treated with and without CKI-7. Cells were grown as described above (a) in the presence or absence of CKI-7 and harvested at LL29 before isolation of flagella. Proteins from the MMA fraction of the flagellum (25 *μ*g each lane) were separated by 9% SDS-PAGE along with a molecular mass standard and immunoblotted with specific antibodies against phosphoSer according to Section 2. Changes in the phosphorylation status of proteins after CKI-7 treatment are indicated by “+” and “−” signs, respectively. (d) Swimming velocity of 137c cells in the absence (−CKI-7) or presence of CK1 inhibitor (+CKI-7). Cells were grown at 23°C in a LD cycle. Measurements of swimming velocity were done with a hemocytometer and a differential interference contrast microscope with a total magnification of 400 including a personal computer with a video recording system to measure displacement versus time (*n* = 10). Error bars represent the SEM.

CK1 was found in the proteome of the flagellum [4] and was also shown immunologically to be enriched in flagella in wild-type strain SAG 73.72 [17]. For the comparative phosphoproteome analysis, flagella were isolated from strain 137c along with the dibucaine method [11]. We first examined the enrichment of CK1 in flagella of 137c using the applied conditions by immunodetection along with anti-CK1 antibodies (Figures 1(a) and 1(b)). Levels of CK1 were significantly enriched in the flagella fraction, especially compared to cell bodies lacking flagella. Thus, the procedure used for identification of the phosphoproteome maintains the enrichment of CK1 in flagella and is thus suited to screen for its targets.

In the next step, we examined to what degree the CK1-specific inhibitor, CKI-7 [24], which was already used for studying CK1 in *C. reinhardtii* [15], influences the phosphorylation pattern of flagellum proteins. Therefore, we grew cells with and without CKI-7 treatment, respectively, and compared the flagellum phosphoproteins from both aliquots by immunodetection with antiphosphoSer antibodies (Figure 1(c)). As expected, several phosphorylated protein bands were reduced to a significant extent or absent in the CKI-7-inhibited cells (Figure 1(c), labeled with “−”). At the same time, some phosphoprotein bands were stronger (Figure 1(c), labeled with “+”). These data show that inhibition of CK1 has a dual effect. On the one hand, the phosphorylation of CK1 targets drops strongly down or is fully stopped by its inhibition; on the other hand, inactive CK1 seems to lead to the activation of other kinases resulting in the phosphorylation of other proteins.

As mentioned before, flagellum kinases affect motility. We also studied if the inhibition by CKI-7 results in changes in swimming velocity. To analyze the swimming behavior, we compared the swimming velocity of the *C. reinhardtii *strain 137c with cells that were cultivated with CKI-7 as described (see Section 2). Cells were spotted on a hemocytometer and the swimming velocity was measured using a differential interference contrast microscope including a personal computer with a video recording system (see Section 2). The assay revealed that the swimming speed of CK1-inhibited cells is significantly lower (75.6 *μ*m/s; ±4,1 SEM) compared to untreated cells (122.2 *μ*m/s; ±2.5 SEM) (Figure 1(d)). These data show that CK1-mediated phosphorylation events in flagella influence motility and swimming speed of *C. reinhardtii* cells.

### 3.2. The Flagellum Phosphoproteome of CKI-7-Treated Cells

The targets of CK1 in the flagellum are of high interest with regard to flagella formation as well as for clock control events. They are largely unknown. An exception is IC138 that is suggested as a direct target of CK1 based on experimental data (summarized in [25]). 

In a next step, the direct and indirect targets of CK1 were analyzed by a functional proteome approach. For that purpose, we compared the already existing phosphoproteome [11] with one investigated exactly under the same conditions with the single exception that CK1 is inhibited. Since strong silencing of CK1 by RNAi results in defects in flagella formation, flagellum material cannot be obtained in a significant amount from such strains [17]. Therefore, inhibition of CK1 with CKI-7 was used. Cells were grown under a light-dark cycle and the inhibitor was added for a 29 h period right at the moment when the cells were released to constant dim light. LL29 was also used as harvesting time point in the previous analysis [11]. 

We avoided to add high amounts of ATP to isolated flagella and to postincubate them at elevated temperatures to induce kinase activities *in vitro*, as done in another study [12]. We found that this treatment leads to severe phosphorylation events that include most likely phosphorylation steps that would not take place *in vivo* under physiological conditions See Supplemental Figures 1(a), 1(b) in Supplementary Material available online at doi: 1155/2012/581460. 

 The further analysis of the phosphoproteome in CKI-7-treated cells was carried out with the same procedure and criteria as applied before for the flagellum phosphoproteome [11]. Three independent isolations of flagella of CKI-7-inhibited cells were carried out and subjected to phosphopeptide purification along with liquid chromatography mass spectrometry (for details, see [11]). Previously identified phosphopeptides or phosphorylation sites within a phosphopeptide ( listed in Table S1 in [11]) that had not been detected in any of the three analyses were considered to be either direct or indirect targets of CK1. The phosphoproteins to which these phosphopeptides belong are listed in Table 1. Novel phosphopeptides belonging to novel phosphoproteins that had not been identified in the former analysis and additional phosphopeptides or phosphorylation sites of already identified phosphoproteins are listed in Table 2. Details about all newly identified peptides and phosphorylation sites can be found in Supplemental Table S1. In three cases, (TEKTIN, FAP18, and FAP262), all previous identified phosphorylation sites were detected again, but in some phosphopeptides with different combinatory phosphorylation patterns (data not shown).

**Figure 2:**
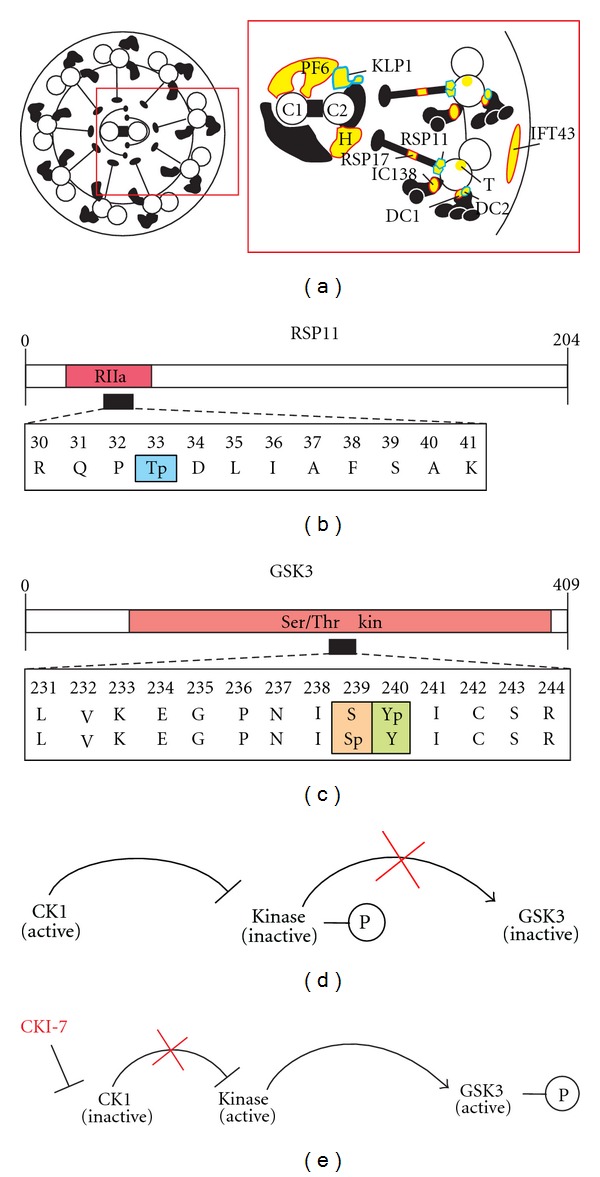
Analysis of CK1 targets in the flagellum. (a) Diagram of flagellum phosphoproteins in wild-type and CK1-inhibited cells. A cross-section of a flagellum from *C. reinhardtii* (left panel) and a more detailed view (red rectangle) are shown according to [11]. Structural phosphoproteins in CKI-7-inhibited cells, and such with a reduced number of phosphorylation sites are indicated in yellow color with a red frame. Novel phosphopeptides of structural proteins or additional phosphorylation sites of known phosphopeptides from structural phosphoproteins that were identified in the CKI-7-inhibited proteome are indicated by yellow color with a blue frame. Structural proteins with previously identified phosphopeptides, whose phosphorylation sites were detected again, are indicated in yellow without frame. Abbreviations are: C1 central pair projection (C1P), C2 central pair projection (C2P), PF6 protein (PF6), Hydin (H), Radial Spoke Protein17 (RSP17); Outer Dynein Arm Docking Complex (DC); Inner Dynein Arm Intermedite Chain138 (IC138), Tectin (T) as well as an Intraflagellar Transport Protein43 (IFT43). (b) and (c) Positions of identified phosphopeptides in the predicted domains of RSP11 (b) and GSK3 (c). Identified phosphopeptides are indicted by black boxes. The amino acid positions are mentioned. “p” indicates *in vivo* phosphorylation sites. RIIa, regulatory subunit of cAMP-Dependent Protein Kinase A; Ser/Thr Kin, Ser/Thr protein kinase catalytic domain. (d) and (e) Hypothetical model of GSK3 de-/activation via reversible phosphorylation triggered by CK1. (d) Regulatory signaling involves an additional kinase. The noninhibited active form of CK1 inactivates another still unknown kinase by phosphorylation. This kinase is needed in its active nonphosphorylated form for activating GSK3. (e) If CK1 is inhibited by CKI-7, the unknown kinase is not phosphorylated and thus active. This active kinase phosphorylates in turn GSK3, which is then activated.

In the CKI-7-treated cells, phosphopeptides from 14 phosphoproteins were missing (Table 1). Four additional phosphoproteins were identified again but with a reduced number of phosphorylation sites. These are labeled by indices along with the missing sites in Table 1. Among these 18 phosphoproteins, six known structural proteins are present including IC138 that was suggested to be a direct target of CK1 [26]. All missing structural phosphoproteins as well as those with a reduced number of phosphorylation sites are indicated in yellow color with a red frame in Figure 2(a). Moreover, seven FAPs with conserved domains are affected in the CKI-7-treated phosphoproteome as well as five FAPs without any conserved domains.

Also novel phosphopeptides or additional phosphorylation sites of known phosphopeptides were identified in the proteome of CKI-7-treated cells, suggesting that CK1 is part of a signaling network in the flagellum. They belong to either 15 new phosphoproteins or six already known phosphoproteins (Table 2, Supplemental Table 1). Among them, some structural components are present, indicated by yellow color with a blue frame in Figure 2(a). Thereby, Radial Spoke Protein 11 (RSP11) is of special interest. It has an RIIa domain, which is a regulatory subunit of cAMP Dependent Protein Kinase A (PKA) and bears a phosphorylation site (Figure 2(b)). Two other kinases were also found in this category. One of them is Glycogen Synthase Kinase 3 (GSK3). The level of active GSK3 is postulated to be regulated via phosphorylation of a conserved Tyr correlating with flagellar length [27]. Exactly this Tyr that is situated in the Ser/Thr kinase domain of GSK3 is phosphorylated as well as a Ser in its surroundings (Supplemental Table 1; Figure 2(c)). Notable GSK3 is also clock relevant, for example, in *Drosophila* [28]. Moreover, a Mitogen Activated Kinase, MAK7, was found with additional phosphorylation sites.

## 4. Discussion

The identification of targets of CK1 in the flagellum will help understanding flagella formation as well as clock control events related to flagella [17–19]. The fact that several phosphorylated flagellar protein bands disappear in CKI-7-treated cells suggests that CK1 has multiple targets in the flagellum. Among the 32 phosphoproteins of the flagellum, 14 were missing in the flagellum phosphoproteome when the CKI-7 inhibitor was used or represented with a reduced number of phosphorylation sites (four cases, Table 1). Missing phosphorylation sites cannot be automatically considered as direct targets of CK1. It could be that the phosphorylation of an amino acid residue by CK1 represents a trigger that then allows a consequent phosphorylation of another amino acid residue in the surroundings by another kinase. An example for consequent phosphorylation steps of different kinases is mentioned below and involves PKA, GSK3, and CK1. Also, CK1 may activate or deactivate another kinase by reversible phosphorylation. In the current study, the previously identified kinases along with their phosphorylation sites were found again [11]. Only in case of FAP262 that bears a Ser/Thr kinase domain, a different combinatory phosphorylation pattern was observed, which might be relevant. But it could also be that some of the missing phosphoproteins in the FAP category whose functions are not known may have kinase activity. Networks that consist of interconnected kinases along with protein phosphatases are not unusual in signaling. In line with this, we found also 21 new phosphoproteins along with novel phosphopeptides or phosphorylation sites, including three kinase-related proteins. The presence of new phosphorylation sites in flagella of CKI-7-inhibited cells was already predictable from the appearance of novel flagellar phosphoprotein bands detected with anti-phosphoSer antibodies (Figure 1(c)). In this category, we identified two phosphoproteins involved in carbohydrate and amino acid metabolism, respectively (Table 2). One of them, phosphoglucomutase, catalyzes the bidirectional conversion of glucose-1-phosphate to glucose-6-phosphate. Glucose-1-phosphate can be transferred into glycolysis by this way. The flagellum contains all enzymes of the late glycolytic pathway; they are able to generate ATP for direct use in the flagellum [4]. In mammals, the activity of phosphoglucomutase is regulated by phosphorylation [29]. The other metabolically relevant enzyme in this category is S-adenosylmethionine synthetase, a key enzyme of methionine metabolism. In rat liver, the activity of the S-adenosylmethionine synthetase is regulated by Protein Kinase C [30]. 

One of the direct targets of CK1 was suggested to be IC138, the Inner Dynein Arm I1 Intermediate Chain 138. It was shown that phosphorylation of IC138 correlates with the inhibition of dynein activity and that PKA beside CK1 as well as the Protein Phosphatases PP2A and PP1 are involved there (summarized in [26]). IC138 was identified in CK1 active cells with one phosphopeptide that is situated at its N-terminus including variable phosphorylation sites [11]. None of these phosphorylation sites were detected after CKI-7 treatment, underlining that IC138 is a direct and/or indirect target of CK1. A pharmacological analysis using CKI-7 revealed the impact of CK1 on IC138 phosphorylation [14]. This mechanism authorizes CK1 to regulate dynein activity and control flagellum motility. Also an analysis of mutants lacking the IC138 subcomplex revealed strains that swim forward with reduced swimming velocities [31, 32]. Interestingly, the swimming speed of the CKI-7-treated cells was reduced to a similar degree in comparison to the mutant strains that are lacking IC138, suggesting that the generation of flagellum motility is regulated by a CK1-mediated phosphorylation of IC138 as suggested before [14, 15]. 

Another structural phosphoprotein previously identified with two phosphopeptides and variable phosphorylation sites is ODA-DC1. The outer dynein arm docking complex (ODA-DC), which is composed of three proteins, designated DC1, DC2, and DC3, is associated with microtubules and targets the outer dynein arms to its binding site on the flagellum axoneme [33]. In both previously identified phosphopeptides certain phosphorylation sites are missing in CKI-7-inhibited cells (Table 1; indices a, b) pointing out that they are CK1 targets. ODA-DC2 had been also identified in the previous study [11] with one phosphopeptide and variable phosphorylation sites, which were all found again in the current study. But now a novel phosphopeptide with phosphorylation on Ser-278 was present in CKI-7 cells, underlining that CK1 seems to be indirectly involved in regulating further kinases.

Radial spokes represent a major structural feature of 9 + 2 axonemes and they are essential for flagellum beating. Each radial spoke consists of a thin stalk, which is attached to the A-tubule of the axonemal doublet microtubules and a head projecting toward the central apparatus [34]. The radial spoke of *C. reinhardtii *is composed of at least 23 proteins, and not all of them have been characterized at the molecular level [35]. RSP17, which is located in the spoke stalk, was identified in the flagellum phosphoproteome analysis with two different phosphopeptides [11]. The absence of both phosphopeptides in CKI-7-treated cells suggests that RSP17 is at the same time a direct and/or indirect target of CK1. Functional domains in radial spoke proteins reveal their role in mediating signaling pathways. For instance, RSP11 consists of a regulatory subunit (RIIa) of PKA [35]. However, RSP11 lacks the cAMP-binding domains of the RII regulatory subunit. We could identify RSP11 in the CKI-7-treated cells as a new phosphoprotein with one *in vivo* phosphorylation site at Thr-35, which is located directly in the RIIa domain (Figure 2(b)). The interaction between RII and A-kinase anchoring protein motifs (AKAP) can be regulated by phosphorylation of RII [36, 37]. A pharmacological analysis using an inhibitor and the RII regulatory subunits had detected an axonemal PKA activity [38]. But PKA could not be found in the flagellar proteome in contrast to CK1, PP1, and PP2A [4]. Thus, it was hypothesized that *C. reinhardtii *could express a PKA with an unconventional structure [39]. The identified phosphorylation site within the RII subunit of RSP11 may be relevant in this context. 

An additional flagellum kinase is GSK3 whose enzymatic activity is inhibited by lithium causing flagellar elongation [27]. It is known that GSK3 has a Tyr-phosphorylated, active form and is enriched in flagella. GSK3 is associated with the axoneme in a phosphorylation-dependent manner. The level of active GSK3 correlates with flagellar length [27]. We could identify the Tyr-240-phosphorylated GSK3 as well as a Ser-239-phosphorylated alternative in the CKI-7-treated cells (Figure 2(c)), suggesting that inhibition of CK1 causes activation of GSK3. Both *in vivo* phosphorylation sites are located in the catalytic kinase domain, which could play important roles in the regulation of the activity of GSK3 within signaling pathways. Notably, interplay between CK1 and GSK3 is known for Hedgehog signaling pathways [40]. Thereby, the Cubitus Interruptus (Ci-155) transcriptional activator is involved. Ci-155 proteolysis depends on phosphorylation at three sites of PKA. Then, these phosphoSer prime further phosphorylation at GSK3 and CK1 sites. This principle is a good example for consecutive phosphorylation steps of different kinases as mentioned before. 

Several studies have shown that reversible phosphorylation of Tyr causes increases and decreases in GSK3 kinase activity, respectively [41, 42]. For the interplay of CK1 and GSK3 in the *C. reinhardtii* flagella, one can imagine a regulatory mechanism, involving, for example, an additional kinase. In a hypothetical model (Figure 2(d)), the noninhibited, active CK1 inactivates another kinase by phosphorylation, which is responsible for the activation of GSK3 by Tyr-phosphorylation. If CKI-7 inhibits CK1 (Figure 2(e)), the additional kinase can stay active, because it is not phosphorylated by CK1 and consequently GSK3 gets converted to the phosphorylated active form. 

GSK3 plays also an important role in the regulation of circadian systems. Shaggy (SGG), for example, the *Drosophila* homologue of GSK3, is a central player in determining period length in flies by phosphorylation of clock components [43]. In mammals, GSK3 is proposed to phosphorylate Clock (CLK), which is a core transcription factor that is essential for circadian behavior. Phosphorylation of CLK controls its activity and degradation [44]. Especially kinases and phosphatases, which are relevant in regulating circadian clocks in other organisms, are well conserved in *Chlamydomonas* [45]. Interestingly, many output rhythms that can be measured like phototaxis, chemotaxis, and stickiness to glass and mating during the cell cycle involve flagella. It is remarkable that kinases like CK1 or GSK3 as well as phosphatases like PP1 and PP2A are physically located in the axoneme [4, 26]. This underlines the important regulatory function of these components in the flagellum regarding circadian rhythms.

## Supplementary Material

Supplemental Figure 1: Influences on the phosphorylation pattern of flagellum proteins by ATP treatment of isolated flagella.
Supplemental Table 1: Newly identified phosphopeptides or phosphorylation sites of already known phosphopeptides in CKI-7-treated cells.

## Figures and Tables

**Table 1:** Phosphoproteins identified in 137c [11] whose phosphopeptides or phosphorylation sites are missing in CKI-7-treated cells.

Flagellar central pair-associated protein; PF6	
Hydin-like protein; HYD3	
Inner dynein arm I1 intermediate chain; IC138	
Intraflagellar transport protein IFT43	
Outer dynein arm docking complex subunit 1^a,b^; ODA-DC1, ODA3	
Radial spoke protein 17; RSP17	
FAP59^c^; RecF/RecN/SMC N-terminal domain	
FAP116^a,d,e^; microtubule-binding protein MIP-T3 domain	
FAP190^a,f^; sterile alpha motif	
FAP228; callose synthase-like protein; 1,3-beta-glucan synthase component	
FAP230; ankyrin repeats; ion transport protein domain	
FAP254; putative ankyrin-like protein	
FAP288; EF hand	
FAP1^a,g^	
FAP93	
FAP147	
FAP184	
FAP263	

The function of depicted proteins is given as determined by NCBI BLASTp, along with their conserved domains.

^a^Not all previously identified peptides (listed in Table S1 in [11]) are present in the CKI-7-treated cells.

^b^Variants of peptide TISGADTPEEVLAYWEGLK with the phosphorylation sites Thr-345, Ser-347, and Thr-351 as well as variants of peptide ILGYTGSDVEEEEPESEEETEEEANKDDGVVDR with the phosphorylation sites Tyr-697 and Ser-709 are missing.

^c^Predicted functional domains are present only in the Vs3 model.

^d^Vs2 model differs significantly from Vs3 model.

^e^The phosphorylation site Ser-255 in peptide SASPGGEDPLNKSGSAAPK is missing.

^f^Variants of peptide STSSIGGGYSEPVGSDGEGSDAASAKPR with phosphorylation sites on Ser-370, Ser-375 and Ser-379 are missing.

^g^The phosphorylation site Ser-55 in peptide SRGSFQEGQAMVR is missing.

**Table 2:** Additional phosphopeptides/phosphorylation sites in CKI-7-treated cells of either novel phosphoproteins or phosphoproteins that were already identified in 137c [11].

Phosphoproteins only present in CKI-7-treated 137c cells	
Glycogen synthase kinase 3; GSK3	
Kinesin-like protein; Kinesin motor domain, KIF9-like subgroup	
Phosphoglucomutase	
Radial spoke protein 11; RSP11; RIIa domain	
S-Adenosylmethionine synthetase	
FAP139^a^; TIGR02680 domain	
FAP21^a^	
FAP56	
FAP75	
FAP98	
FAP129	
FAP165^a^	
FAP236	
FAP241	
FAP243 (Vs3 FAP183)^a^	

Phosphoproteins found in CKI-7-treated cells with additional phosphopeptide(s) in comparison to 137c	

Outer dynein arm docking complex protein 2; ODA-DC2	
FAP33^a,b^; ankyrin repeats	
FAP154^a^	
FAP217	

Phosphoproteins found in CKI-7-treated cells with the same peptide [11] but with additional phosphorylation site(s)	

FAP39^a^; plasma membrane calcium transporting ATPase	
MAK7^a^; mitogen activated protein kinase 7	

The function of depicted proteins is given as determined by NCBI BLASTp, along with their conserved domains.

^a^Vs2 model differs significantly from Vs3 model.

^b^Predicted functional domains are present only in the Vs3 model.
