# Supplementary material for: Application of Phosphoproteomics to Find Targets of Casein Kinase 1 in the Flagellum of Chlamydomonas
Source: Int J Plant Genomics. 2012 Dec 18;2012:581460. doi: 10.1155/2012/581460 (PMC3536430; doi:10.1155/2012/581460)
Supplement: Supplementary file 1 — Supplemental Figure 1: Influences on the phosphorylation pattern of flagellum proteins by ATP treatment of isolated flagella. Supplemental Table 1: Newly identified phosphopeptides or phosphorylation sites of already known phosphopeptides in CKI-7-treated cells. [file 581460.f1.pdf]

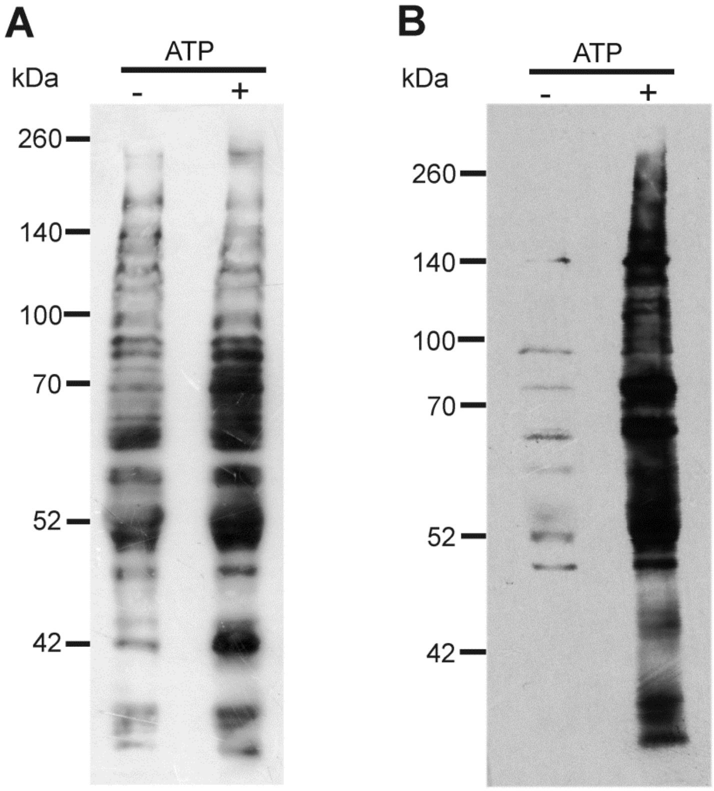

Supplemental Figure 1. Influences on the phosphorylation pattern of flagellum proteins by ATP treatment of isolated flagella. Cells were grown in a LD cycle and then released to dim light (LL) according to Methods. Cells were harvested at LL29 and flagella were isolated and resuspended in a HMD buffer containing phosphatase inhibitors and Nonidet NP-40 according to [11]. The flagella extract was incubated at 25°C for 10 min in the absence (-) or presence (+) of 1 mM ATP according to [12]. Proteins from the flagellum matrix/membrane/axoneme fraction were separated by 9% SDS-PAGE (25 µg per lane) along with a molecular mass standard and immunoblotted with anti-phosphoSer (A) and anti-phosphoThr (B) antibodies, respectively, according to Methods.

Supplemental Table 1. Newly identified phosphopeptides or phosphorylation sites of already known phosphopeptides in CKI-7 treated cells.

Protein-ID Vs2	Protein-ID Vs3	AA / MW	Phosphopeptide along with the number of the first amino acid within its protein	z	Xcorr	Function and/or homologies of predicted proteins determined by NCBI BLASTp
**Phosphoproteins only present in CKI-7 treated 137c cells**						
166046	114692	409 AA / 45.7 kDa	234-GEPNISYpICSR<br>230-VLVKGEPNISYpICSR<br>-> VLVKGEPNISpYICSR	2<br>2<br>2	2.62<br>2.64<br>2.62	ref|XP_001690881.1|, gb|AAT40314.1| glycogen synthase kinase 3 [Chlamydomonas reinhardtii], 0.0; Ser/Thr protein kinase domain, 6.91e-79
167565	186414	776 AA / 82.9 kDa	454-GGDSpAGPSGVGDVDLR	2	3.51	ref|XP_001701617.1|, gb|EDP06592.1|, kinesin-like protein [Chlamydomonas reinhardtii], 0.0; Kinesin motor domain, KIF9-like subgroup, 1.85e-140
165299	81483	600 AA / 64.5 kDa	148-HLYGGLIMSASpHNPGGPENDFGIK<br>-> HLYGGLIMSpASHNPGGPENDFGIK	3<br>3	4.46<br>4.4	ref|XP_001697991.1|,gb|EDO99576.1| phosphoglucomutase [Chlamydomonas reinhardtii], 0.0
170435	182635	204 AA / 21.4 kDa	32-RQPTpDLIAFSAK	2	3.43	ref|XP_001698630.1|, gb|ABC02022.1| radial spoke protein 11 [Chlamydomonas reinhardtii], 8e-115; RIIalpha domain [32]
169475	182408	390 AA / 42.5 kDa	95-VLVHIEEQSPDIGQGVHGMoGTpK<br>-> VLVHIEEQSPDIGQGVHGMGTpK	3<br>3	6.26<br>5.17	ref|XP_001696661.1|, gb|EDP08638.1| S-Adenosylmethionine synthetase [Chlamydomonas reinhardtii], 0.0

171076	191309 [a]	1083 AA / 114.1 kDa	295-YELDDDGNLASpFSR	2	2.67	ref|XP_001694913.1|, gb|EDP02065.1| flagellar associated protein [Chlamydomonas reinhardtii], 0.0; found in the flagellar proteome as FAP139
169308 [b]	34449 [a]	425 AA / 45.8 kDa [c]	37-DALTWNDERPSTpPDDIKK<br>-> DALTWNDERPSpTPDDIKK	3<br>3	4.51<br>3.6	ref|XP_001695261.1|, gb|EDP01969.1| flagellar associated protein [Chlamydomonas reinhardtii], 2e-101; found in the flagellar proteome as FAP21
165688	143938	551 AA / 60.4 kDa	20-VNDPNAPEQALIPTSpPR<br>-> VNDPNAPEQALIPTpSPR	2<br>2	3.72<br>3.17	ref|XP_001703474.1|, gb|EDP06156.1| flagellar associated protein [Chlamydomonas reinhardtii], 0.0; found in the flagellar proteome as FAP56
169508	142494	1264 AA / 125.2 kDa	1087-QSSpAEAAEVAEAEAAAAAAEAQR<br>-> QSpSAEAAEVAEAEAAAAAAEAQR	2<br>2	4.85<br>4.62	ref|XP_001696434.1|,gb|EDP08411.1| flagellar associated protein [Chlamydomonas reinhardtii], 0.0; found in the flagellar proteome as FAP75
157003	147732	522 AA / 59 kDa	129-SpADAPAAGSVPVDWTLPQR	2	2.82	ref|XP_001692676.1|, gb|EDP03695.1| flagellar associated protein [Chlamydomonas reinhardtii], 0.0; found in the flagellar proteome as FAP98
154693	190617	496 AA / 52.6 kDa	97-AFSKTpGGGYWISQR	2	2.76	ref|XP_001693237.1|, gb|EDP03263.1| flagellar associated protein [Chlamydomonas reinhardtii], 0.0; found in the flagellar proteome as FAP129

153321 [b]	193605 [a]	809 AA / 99.5 kDa [c]	20-RRTpDDVINPLDITEAELK	3	5.46	ref|XP_001699413.1|, gb|EDO98659.1|, flagellar associated protein [Chlamydomonas reinhardtii], 1e-56; found in the flagellar proteome as FAP165
165288	192970	457 AA / 49.7 kDa	277-GPFAGTSTYATEYRPRTpAPAYER<br>-> GPFAGTSTYATEYRPRTAPAYpER	3<br>3	4.87<br>4.57	ref|XP_001698000.1|, gb|EDO99585.1| flagellar associated protein [Chlamydomonas reinhardtii], 0.0; found in the flagellar proteome as FAP236
167668	141178	359 AA / 41.2 kDa	30-LVGGTpPGKSSLVDAK	2	3.28	ref|XP_001701817.1|, gb|EDP06792.1| flagellar associated protein [Chlamydomonas reinhardtii], 0.0; found in the flagellar proteome as FAP241
169099	193154 [d]	996 AA / 106.1 kDa	888-TPDTNPLLLASpAPPTpPR	2	3.14	ref|XP_001698225.1|, gb|EDO99510.1| flagellar associated protein [Chlamydomonas reinhardtii], 1e-180; found in the flagellar proteome as FAP243; annotated as FAP183 in Vs3
**Phosphoproteins found in CKI-7 treated cells with additional phosphopeptide(s) in comparison to 137c[e]**						
171763	132719	552 AA / 62.2 kDa	274-ITKGSpWNVGYNK	2	3.56	ref|XP_001701436.1|, gb|EDO97433.1| flagellar outer dynein arm-docking complex protein 2 (ODA1; ODA-DC2) [Chlamydomonas reinhardtii], 0.0
157568	36026 [a]	1329 AA / 138 kDa	38-AHRISpGGGDGALAASGFADATVK	3	5.16	ref|XP_001699049.1|, gb|EDO99103.1| flagellar associated protein [Chlamydomonas reinhardtii], 0.0; found in the flagellar proteome as FAP33; in Vs3 annotated as ANK17 with ankyrin repeats, 3e-15

156262<br>156264 [f]	16685 [a]	2324 AA /<br>244.9 kDa	1767-ALASQTTHLLDDDESpDDEEDEDGAAAAAASK	3	6.45	ref|XP_001696012.1|, gb|EDP00961.1| flagellar associated protein [Chlamydomonas reinhardtii], 2e-61; annotated as FAP49 (156262) and FAP154 (156264) in Vs2; both models were fused to FAP154 in Vs3
			-> ALASQTTpHLLDDDESDDEEDEDGAAAAAASK	3	4.9	
			-> ALASQTpTHLLDDDESDDEEDEDGAAAAAASK	3	4.85	
			-> ALASpQTTHLLDDDESDDEEDEDGAAAAAASK	3	4.75	
153201	146112	1301 AA /<br>133.9 kDa	433-AADDPFLVELTPDGIPIEEATpTCDLRR	3	4.03	ref|XP_001692474.1|, gb|EDP03952.1| flagellar associated protein [Chlamydomonas reinhardtii], 0.0; found in the flagellar proteome as FAP217
			-> AADDPFLVELTPDGIPIEEATTpCDLRR	3	4.02	
**Phosphoproteins found in CKI-7 treated cells with the same peptide (Boesger et al. 2009) but with additional phosphorylation site(s)[g]**						
166958	189266 [d]	1156 AA /<br>124.6 kDa	1137-<u>Sp</u>AGSGSTAPLLIDPSTpPR	2	3.66	ref|XP_001692057.1|, gb|EDP04547.1| plasma membrane calcium-transporting ATPase [Chlamydomonas reinhardtii], 0.0; found in the flagellar proteome as FAP39
			->SAGSpGSTAPLLIDP<u>Sp</u>TPR	2	3.55	
			->SpAGSGSTAPLLIDP<u>Sp</u>TPR	2	3.47	
			->SAGSG<u>Sp</u>TAPLLIDPSTpPR	2	3.09	
			->SAGSG<u>Sp</u>TAPLLIDP<u>Sp</u>TPR	2	2.88	
			->SAGSpGS<u>Tp</u>APLLIDPSTPR	2	2.80	
			->SAGSGS<u>Tp</u>APLLIDPSTpPR	2	2.79	
			->SAGSGS<u>Tp</u>APLLIDP<u>Sp</u>TPR	2	2.58	
168935	58843 [a]	585 AA /<br>63.3 kDa	151-SRPPYTpDYVS<u>Tp</u>R	2	3.20	ref|XP_001697865.1|, gb|EDO99748.1| mitogen-activated protein kinase 7 [Chlamydomonas reinhardtii], 5e-178
			->SRPPYTDYpVS<u>Tp</u>R	2	2.64	
			->SRPPYpTDYVS<u>Tp</u>R	2	2.53	

AA, number of amino acids according to [4]. MW, molecular weight according to (Pazour et al., 2005). z, charge. Sp, phosphorylated Ser or Dehydroalanine. Tp, phosphorylated Thr or Methyldehydroalanine. Yp, phosphorylated Tyr. Mo, oxidized Met. Function and/or homologies of depicted proteins are given as determined by NCBI BLASTp along with their conserved domains and their e-values. If a peptide is marked with an arrow, the peptide above shows identical or overlapping sequences, but the phosphorylation sites may be different. [a] Sequence of Vs3 model differs significantly from sequence of Vs2 model, peptides were present in both. [b] Vs2 model contains unidentified amino acids. [c] AA, MW and the position of the first amino acids of the peptides were calculated according to the Vs3 model. [d] Sequence of Vs3 model differs significantly from sequence of Vs2 model, peptide is only present in Vs2 model. [e] Only additional phosphopeptides are shown. Former identified peptides are listed in Table S1 in [11]. [f] Vs2 models were combined to one model in Vs3. [g] Only phosphopeptides containing novel phosphorylation sites are shown. Novel phosphorylation sites are underlined.
